# Supplementary material for: Immunohistochemical Expression of Epithelial Cell Adhesion Molecule (EpCAM) in Salivary Gland Cancer: Correlation with the Biological Behavior
Source: Diagnostics (Basel). 2023 Aug 11;13(16):2652. doi: 10.3390/diagnostics13162652 (PMC10453306; doi:10.3390/diagnostics13162652)
Supplement: Supplementary file 1 [file diagnostics-13-02652-s001.zip › diagnostics-2541052-supplementary.pdf]

	n	Intensity Score (IS)				p-value	n	Proportion Score (PS)					p-value	n	Total Immunostaining Score (TIS)				p-value
		0	1	2	3			0	1	2	3	4			0	1,4	6,8	9,12	
		no staining	weak staining	moderate staining	strong staining			0% positively stained tumor cells	<10% positively stained tumor cells	10-50% positively stained tumor cells	51-80% positively stained tumor cells	>80% positively stained tumor cells			no staining	weak staining	moderate staining	strong staining	
**Histopathological subtypes of SGC**																			
Adenocarcinoma (NOS)	4	0 (0.0)	3 (75.0)	1 (25.0)	0 (0.0)		4	0 (0.0)	3 (75.0)	1 (25.0)	0 (0.0)	0 (0.0)		4	0 (0.0)	4 (100.0)	0 (0.0)	0 (0.0)	
Adenoid cystic carcinoma (AdCC)	18	0 (0.0)	1 (5.6)	12 (66.7)	5 (27.8)		18	0 (0.0)	1 (5.6)	5 (27.8)	9 (50.0)	3 (16.7)		18	0 (0.0)	6 (33.3)	7 (38.9)	5 (27.8)	
Mucoepidermoid carcinoma (MEC)	20	4 (20.0)	4 (20.0)	8 (40.0)	4 (20.0)		20	4 (20.0)	4 (20.0)	7 (35.0)	1 (5.0)	4 (20.0)		20	4 (20.0)	11 (55.0)	1 (5.0)	4 (20.0)	
Polymorphous adenocarcinoma (PAC)	9	2 (22.2)	6 (66.7)	1 (11.1)	0 (0.0)		9	2 (22.2)	6 (66.7)	1 (11.1)	0 (0.0)	0 (0.0)		9	2 (22.2)	6 (66.7)	1 (11.1)	0 (0.0)	
Epithelial-myoepithelial carcinoma	5	1 (20.0)	2 (40.0)	2 (40.0)	0 (0.0)	**<0.001**	5	1 (20.0)	3 (60.0)	0 (0.0)	1 (20.0)	0 (0.0)	**<0.001**	5	1 (20.0)	3 (60.0)	1 (20.0)	0 (0.0)	**0.001**
Acinic cell carcinoma (AcCC)	2	1 (50.0)	1 (50.0)	0 (0.0)	0 (0.0)		2	1 (50.0)	1 (50.0)	0 (0.0)	0 (0.0)	0 (0.0)		2	1 (50.0)	1 (50.0)	0 (0.0)	0 (0.0)	
Salivary duct carcinoma	5	0 (0.0)	0 (0.0)	0 (0.0)	5 (100.0)		5	0 (0.0)	0 (0.0)	0 (0.0)	0 (0.0)	5 (100.0)		5	0 (0.0)	0 (0.0)	0 (0.0)	5 (100.0)	
Carcinoma-ex pleomorphic adenoma	2	0 (0.0)	0 (0.0)	1 (50.0)	1 (50.0)		2	0 (0.0)	0 (0.0)	0 (0.0)	2 (100.0)	0 (0.0)		2	0 (0.0)	0 (0.0)	1 (50.0)	1 (50.0)	
**Age**																			
≤50	21	3 (14.3)	6 (28.6)	8 (38.1)	4 (19.0)	0.945	21	3 (14.3)	6 (28.6)	5 (23.8)	3 (14.3)	4 (19.0)	0.942	21	3 (14.3)	11 (52.4)	3 (14.3)	4 (19.0)	0.903
>50	44	5 (11.4)	11 (25.0)	17 (38.6)	11 (25.0)		44	5 (11.4)	12 (27.3)	8 (18.2)	10 (22.7)	9 (20.5)		44	5 (11.4)	20 (45.5)	8 (18.2)	11 (25.0)	
**Gender**																			
Male	30	1 (3.3)	9 (30.0)	13 (43.3)	7 (23.3)	0.225	30	1 (3.3)	9 (30.0)	8 (26.7)	6 (20.0)	6 (20.0)	0.284	30	1 (3.3)	17 (56.7)	5 (16.7)	7 (23.3)	0.200
Female	35	7 (20.0)	8 (22.9)	12 (34.3)	8 (22.9)		35	7 (20.0)	9 (25.7)	5 (14.3)	7 (20.0)	7 (20.0)		35	7 (20.0)	14 (40.0)	6 (17.1)	8 (22.9)	
**Location**																			
· Major salivary glands																			
Parotid gland	24	1 (4.2)	7 (29.2)	5 (20.8)	11 (45.8)		24	1 (4.2)	7 (29.2)	1 (4.2)	6 (25.0)	9 (37.5)	0.051	24	1 (4.2)	8 (33.3)	4 (16.7)	11 (45.8)	
Submandibular gland	4	1 (25.0)	0 (0.0)	3 (75.0)	0 (0.0)	**0.027**	4	1 (25.0)	0 (0.0)	1 (25.0)	2 (50.0)	0 (0.0)		4	1 (25.0)	1 (25.0)	2 (50.0)	0 (0.0)	0.116
Sublingual gland	2	0 (0.0)	0 (0.0)	2 (100.0)	0 (0.0)		2	0 (0.0)	0 (0.0)	1 (50.0)	1 (50.0)	0 (0.0)		2	0 (0.0)	1 (50.0)	1 (50.0)	0 (0.0)	
· Minor salivary glands																			
Palate	21	3 (14.3)	7 (33.3)	9 (42.9)	2 (9.5)		21	3 (14.3)	8 (38.1)	6 (28.6)	3 (14.3)	1 (4.8)		21	3 (14.3)	14 (66.7)	2 (9.5)	2 (9.5)	
Tongue/Floor of mouth	4	0 (0.0)	0 (0.0)	3 (75.0)	1 (25.0)		4	0 (0.0)	0 (0.0)	2 (50.0)	1 (25.0)	1 (25.0)		4	0 (0.0)	2 (50.0)	1 (25.0)	1 (25.0)	0.416
Upper lip	2	1 (50.0)	0 (0.0)	1 (50.0)	0 (0.0)	0.433	2	1 (50.0)	0 (0.0)	0 (0.0)	0 (0.0)	1 (50.0)	0.390	2	1 (50.0)	0 (0.0)	0 (0.0)	0 (0.0)	
Lower lip	2	0 (0.0)	0 (0.0)	1 (50.0)	1 (50.0)		2	0 (0.0)	0 (0.0)	1 (50.0)	0 (0.0)	1 (50.0)		2	0 (0.0)	1 (50.0)	1 (50.0)	1 (50.0)	
Retromolar mucosa	3	1 (33.3)	2 (66.7)	0 (0.0)	0 (0.0)		3	1 (33.3)	2 (66.7)	0 (0.0)	0 (0.0)	0 (0.0)		3	1 (33.3)	2 (66.7)	0 (0.0)	0 (0.0)	
Buccal mucosa	3	1 (33.3)	1 (33.3)	1 (33.3)	0 (0.0)		3	1 (33.3)	1 (33.3)	1 (33.3)	0 (0.0)	0 (0.0)		3	1 (33.3)	2 (66.7)	0 (0.0)	0 (0.0)	
**Recurrence**																			
**Local recurrence**																			
Present	6	0 (0.0)	0 (0.0)	1 (16.7)	5 (83.3)	**0.006**	6	0 (0.0)	0 (0.0)	0 (0.0)	12 (20.3)	5 (83.3)	**0.004**	6	0 (0.0)	0 (0.0)	1 (16.7)	5 (83.3)	**0.003**
Absent	59	8 (13.6)	17 (28.8)	24 (40.7)	10 (16.9)		59	8 (13.8)	18 (30.5)	13 (22.0)	1 (16.7)	8 (13.6)		59	8 (13.6)	31 (52.5)	10 (16.9)	10 (16.9)	
**Distant metastasis**																			
Present	24	1 (4.2)	0 (0.0)	12 (50.0)	11 (45.8)	**<0.001**	24	1 (4.2)	0 (0.0)	3 (12.5)	9 (37.5)	11 (45.8)	**<0.001**	24	1 (4.2)	3 (12.5)	9 (37.5)	11 (45.8)	**<0.001**
Absent	41	7 (17.1)	17 (41.5)	13 (31.7)	4 (9.8)		41	7 (17.1)	18 (43.9)	10 (24.4)	4 (9.8)	2 (4.9)		41	7 (17.1)	28 (68.3)	2 (4.9)	4 (9.8)	
**Perineural Invasion**																			
Present	29	3 (10.3)	0 (0.0)	13 (44.8)	13 (44.8)	**<0.001**	29	3 (10.3)	0 (0.0)	5 (17.2)	8 (27.6)	13 (44.8)	**<0.001**	29	3 (10.3)	5 (17.2)	8 (27.6)	13 (44.8)	**<0.001**
Absent	36	5 (13.9)	17 (47.2)	12 (33.3)	2 (5.6)		36	5 (13.9)	18 (50.0)	8 (22.2)	5 (13.9)	0 (0.0)		36	5 (13.9)	26 (72.2)	3 (8.3)	2 (5.6)	
**Vascular invasion**																			
Present	27	1 (3.7)	1 (3.7)	13 (48.1)	12 (44.4)	**<0.001**	27	1 (3.7)	1 (3.7)	8 (29.6)	5 (18.5)	12 (44.4)	**<0.001**	27	1 (3.7)	9 (33.3)	5 (18.5)	12 (44.4)	**0.002**
Absent	38	7 (18.4)	16 (42.1)	12 (31.6)	3 (7.9)		38	7 (18.4)	17 (44.7)	5 (13.2)	8 (21.1)	1 (2.6)		38	7 (18.4)	22 (57.9)	6 (15.8)	3 (7.9)	
**Bone invasion**																			
Present	9	1 (11.1)	1 (11.1)	5 (55.6)	2 (22.2)	0.713	9	1 (11.1)	1 (11.1)	5 (55.6)	1 (11.1)	1 (11.1)	0.119	9	1 (11.1)	6 (66.7)	0 (0.0)	2 (22.2)	0.503
Absent	56	7 (12.5)	16 (28.6)	20 (35.7)	13 (23.2)		56	7 (12.5)	17 (30.4)	8 (14.3)	12 (21.4)	12 (21.4)		56	7 (12.5)	25 (44.6)	11 (19.6)	13 (23.2)	
**Muscle invasion**																			
Present	35	6 (17.1)	6 (17.1)	15 (42.9)	8 (22.9)	0.255	35	6 (17.1)	6 (17.1)	11 (31.4)	4 (11.4)	8 (22.9)	**0.013**	35	6 (17.1)	14 (46.7)	4 (11.4)	8 (22.9)	0.453
Absent	30	2 (6.7)	11 (36.7)	10 (33.3)	7 (23.3)		30	2 (6.7)	12 (40.0)	2 (6.7)	9 (30.0)	5 (16.7)		30	2 (6.7)	17 (48.6)	7 (23.3)	7 (23.3)	

Figure S1. EpCAM expression by IS, TIS and clinicopathological parameters in salivary gland cancer (SCG).
